# The use of UV-C radiation for terminal disinfection of pathogenic Gram-negative rods: a pilot study

**DOI:** 10.1017/ash.2023.514

**Published:** 2023-12-21

**Authors:** Joseph Tholany, Hiroyuki Suzuki, Amy R. Frank, Steven H. Bryant, Cassie Cunningham Goedken, Daniel Suh, Michael S. Stevens, Stacey M. Hockett Sherlock, Eli N. Perencevich

**Affiliations:** 1 Center for Access & Delivery Research & Evaluation (CADRE), Iowa City Veterans Affairs Health Care System, Iowa City, IA, USA; 2 Division of Infectious Diseases, Department of Internal Medicine, University of Iowa, Iowa City, IA, USA; 3 Iowa City Veterans Affairs Health Care System, Iowa City, IA, USA; 4 Department of Internal Medicine, University of Iowa, Iowa City, IA, USA

## Abstract

In this controlled study, we found that exposure to ultraviolet-C (UV-C) radiation was able to arrest the growth of selected pathogenic enteric and nonfermenting Gram-negative rods. Further studies are needed to confirm the clinical efficacy and determine optimal implementation strategies for utilizing UV-C terminal disinfection.

## Introduction

Reducing nosocomial transmission of pathogenic bacteria remains an important goal for hospitals. Hospital surfaces can become contaminated by patients carrying multidrug-resistant pathogens, which can then become a source of hospital-acquired infections (HAIs) for other patients especially at high-touch surfaces^1,2^ Terminal cleaning and disinfection may prevent such transmission and reduce HAIs.^3^

Terminal disinfection strategies like ultraviolet C (UV-C) radiation present an opportunity to reduce hospital bacterial contamination in conjunction with standard cleaning techniques.^4,^ ^5^ UV-C radiation causes breaks in microbial DNA, decreasing the ability of bacteria to replicate.^6^ A randomized controlled trial (RCT) demonstrated decreased rates of infection by the same causative pathogen of 10%–30% when patients were admitted to the same room that was disinfected using UV-C in addition to standard practices compared to patients who were admitted to the same room that was disinfected with standard practices alone, with bleach alone, or with UV-C and bleach.^7^

UV-C radiation has mostly been studied for *C. difficile* or Gram-positive multidrug-resistant pathogens including methicillin-resistant *S. aureus* and vancomycin-resistant enterococci. Some studies have shown that UV-C radiation successfully suppressed the growth of Gram-negative rods (GNRs) such as *A. baumannii* ^4,7,8^ and carbapenem-resistant enterobacterales.^9^ However, studies evaluating clinical outcomes offer conflicting results. One before–after study showed a decreased incidence of HAIs including those caused by *K. pneumoniae* and *A. baumannii* after implementing UV-C terminal disinfection.^8^ The RCT failed to show a significant decrease in *A. baumannii* transmission, although the incidence rate of infection may have been too low to detect a difference.^7^ A prior stepped-wedge time-series analysis done within the Veterans Health Administration (VHA) suggested that UV-C terminal disinfection was associated with decreased rates of nosocomial GNR bacteremia, which was mainly due to nonendogenous nonfermenters (e.g., *P. aeruginosa*, *A. baumannii*) as compared to enteric fermenters (e.g., *E. coli*, *K. pneumoniae*).^10^ While this epidemiologic study suggested a difference in the clinical impact of UV-C radiation on HAIs caused by different types of GNRs, it is still unclear whether the effect of UV-C radiation truly varies among these GNRs.

We conducted a controlled study to evaluate the effect of UV-C radiation on the growth of pre-inoculated GNRs including both enterics and nonfermenters.

## Methods

A suspension was prepared with one of four common wild-type GNRs: *E. coli*, *K. pneumoniae*, *P. aeruginosa*, and *A. baumannii*. Each suspension was prepared to the 0.5 MacFarland standard in 0.9% saline and was diluted by a factor of 10,000. Ten µL of this suspension was then spread evenly on a plate with eosin methylene blue agar using a nonabsorbent spreader.

**Figure 1.**
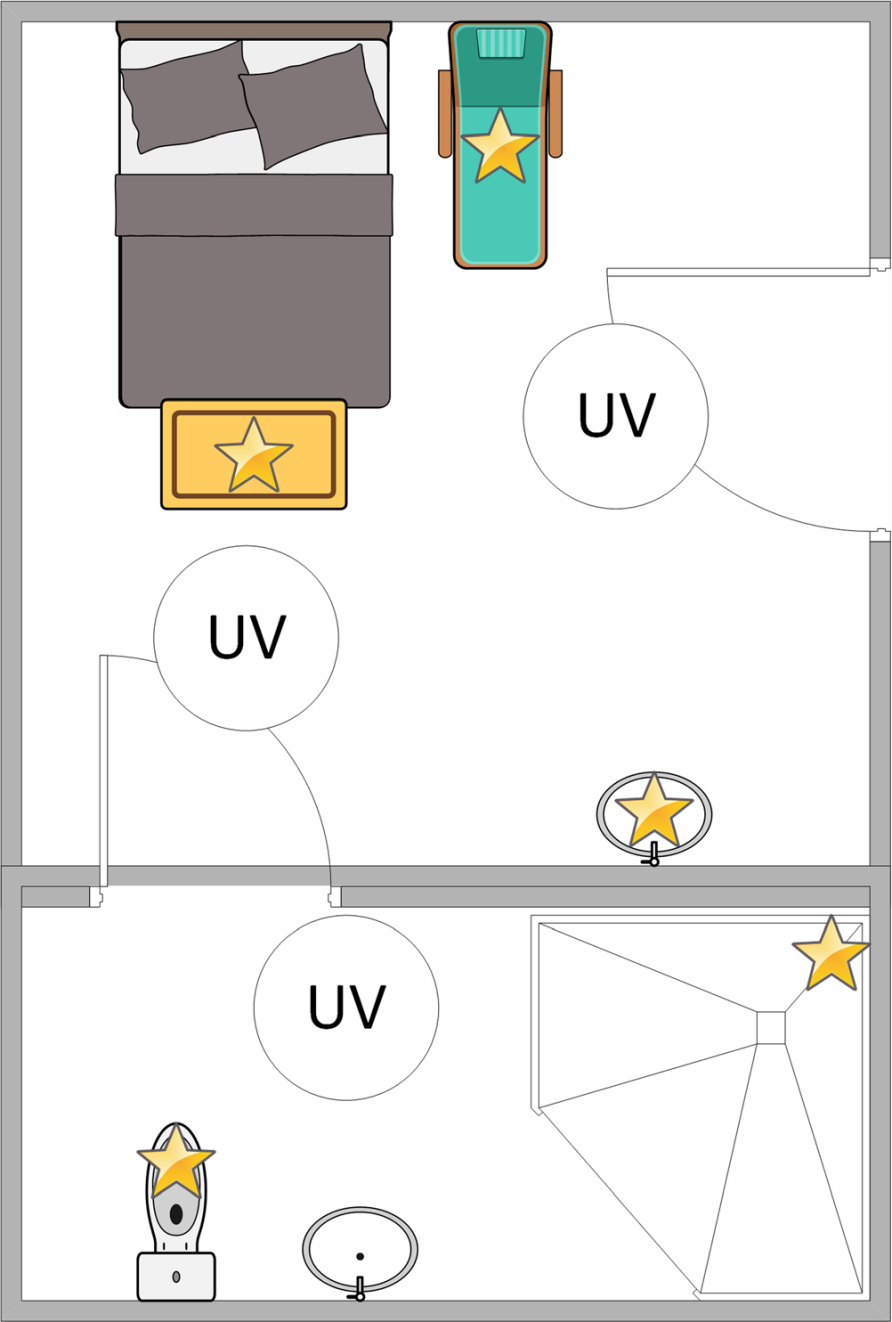
Sample room layout. Four plates, one with each organism (*E. coli*, *K. pneumoniae*, *P. aeruginosa*, *A. baumannii*), were placed on five high-touch sites (yellow stars). Three UV-C machine towers (white circles) were then placed in the room. All blinds and doors were closed prior to running the UV-C machine.

The plates were then placed in an unoccupied standard acute care hospital room at one of five high-touch surface sites: the bedside table, the bedside chair seat, the patient room sink, the bathroom toilet bowl, and the bathroom shower rail. No sites were directly inoculated. The Helios UV-C machine’s three towers (Surfacide, Waukesha WI 53186) were brought into the room: two in the patient room and one in the bathroom (Figure 1). For each plate, the distance from the nearest UV-C tower was recorded; all the plates had direct exposure to at least one UV-C tower. The plates were then exposed to UV-C for a period as determined by the UV-C machine. The UV-C dose uptake was measured when available using a UV-C test card (Clorox, Oakland CA 94612) placed beside the plates. The cards were observed for color changes to verify adequate UV-C exposure. A set of control plates was placed outside of the UV-C exposure area. This process was repeated in multiple rooms over a period of three days. The plates were then returned to the laboratory for incubation at 35°C at room air for 48 hours, with the number of colony-forming units (CFUs) being recorded after 24 hours and 48 hours of incubation. This process was repeated using multiple rooms for a total of seven times over three days. Standard descriptive statistics were used.

## Results

In total, 140 isolates exposed to UV-C radiation and twelve control plates were evaluated for growth. The median distance of the exposed plates from the nearest UV-C tower was 119.4 cm (interquartile range [IQR] 85.1–147.3 cm). The median UV-C machine runtime was 29 minutes (IQR 21–33 minutes). Of the 35 test cards set out, 34 (97.1%) had a color change suggesting adequate UV-C exposure.

The average number of CFUs for all the exposed plates after 48 hours was 0.4 and for the control plates was 87.4 (2.35 log reduction; Table 1). Of the 140 exposed isolates, six (4.3%) experienced growth after 24 hours and ten (7.1%) after 48 hours; all twelve of the controls experienced growth at 48 hours. For the ten exposed plates that experienced growth, four of the isolates were *A. baumannii* (2.9%), and two each were *E. coli* (1.4%), *K. pneumoniae* (1.4%), and *P. aeruginosa* (1.4%). These 10 plates were a median of 161.9 cm (IQR 127.0–227.3 cm) from the nearest UV-C tower with a median runtime of 33 minutes (IQR 27–42 minutes). Seven of the ten were on the shower rail, one each was on the patient room sink, bedside table, and toilet bowl. The average number of CFUs for the exposed plates with growth after 48 hours compared to their control plates was 5.5 and 74.8, respectively (1.13 log reduction).

**Table 1.** Plate growth results following UV-C exposure

Organism	Number of rooms	Number of exposed plates	Number of control plates	Median distance from UV-C tower in cm (IQR)	Median UV-C machine runtime in minutes (IQR)	Mean CFUs at 48 hours post-UV-C	Mean control CFUs at 48 hours	Log reduction	Percentage reduction
**All**	**7**	**140**	**12**	**119.4 (85.1–147.3)**	**29 (21–33)**	**0.4**	**87.4**	**2.35**	**99.54**
*E. coli*	7	35	3	119.4 (83.8**–**151.8)	29 (21**–**33)	0.7	65.3	1.96	98.93
*K. pneumoniae*	7	35	3	119.4 (82**–**137.8)	29 (21**–**33)	0.1	69.3	3.08	99.86
*P. aeruginosa*	5	35	3	119.4 (95.3**–**148.6)	27 (21**–**29)	0.2	120.3	2.78	99.83
*A. baumannii*	5	35	3	114.3 (83.8**–**146.7)	32 (27**–**42)	0.6	94.7	2.20	99.37
Positive growth	4	10	8	161.9 (127.0**–**227.3)	33 (27**–**42)	5.5	74.8	1.13	92.65
Inadequate exposure	1	4	4	143.5 (126.4**–**161.9)	33	11.5	63	0.74	81.75

Three of the positive plates on the shower rail were also associated with the single test card that showed inadequate UV-C exposure (*E. coli*, *P. aeruginosa*, and *A. baumannii*), while one plate from the same set had no growth (*K. pneumoniae*). These plates were a median of 143.5 cm (IQR 126.4–161.9) from the nearest UV-C tower; the average number of CFUs for these plates after 48 hours compared to their controls was 11.5 and 63, respectively (0.74 log reduction).

## Discussion

In this controlled study, the use of UV-C radiation was observed to successfully terminate the growth of both enteric and nonfermenting GNRs, without any notable differences between the growths of the different species at varying distances.

These observations are concordant with previous studies suggesting that UV-C radiation is efficacious for terminal disinfection for GNR infections.^4,9^ The previous VHA study noted a less robust reduction in hospital-onset bacteremia caused by enteric GNRs since these pathogens arose from endogenous sources rather than from hospital surface transmission.^10^ This is unlike the nonfermenters, which are more commonly nosocomial pathogens causing bacteremia. UV-C disinfection likely has a less robust effect on enteric GNRs, but this should not preclude its use.

Our study has limitations. First, this was a small, unblinded, experimental study even though an empty hospital room was used. Bacterial growth was measured on culture plates and was not from inoculated surfaces. Second, measurement of UV-C radiation was not routinely available. The UV-C test card used to assess the exposure level was not a quantitative measure. Therefore, the assessment of appropriate UV-C exposure may have been subject to bias.

In conclusion, the use of UV-C radiation as an adjunct to standard cleaning procedures may be useful in decreasing hospital contamination and infections by GNRs. Its routine use for terminal disinfection should be considered, though further studies are needed to confirm its clinical effectiveness and optimal implementation strategies.
